# Reconsidering Cluster Bias in Multilevel Data: A Monte Carlo Comparison of Free and Constrained Baseline Approaches

**DOI:** 10.3389/fpsyg.2018.00255

**Published:** 2018-03-02

**Authors:** Nigel Guenole

**Affiliations:** Goldsmiths, University of London, London, United Kingdom

**Keywords:** multilevel confirmatory factor analysis, cluster bias, measurement invariance, isomorphism, homology, Monte Carlo

## Abstract

The test for item level cluster bias examines the improvement in model fit that results from freeing an item's between level residual variance from a baseline model with equal within and between level factor loadings and between level residual variances fixed at zero. A potential problem is that this approach may include a misspecified unrestricted model if any non-invariance is present, but the log-likelihood difference test requires that the unrestricted model is correctly specified. A free baseline approach where the unrestricted model includes only the restrictions needed for model identification should lead to better decision accuracy, but no studies have examined this yet. We ran a Monte Carlo study to investigate this issue. When the referent item is unbiased, compared to the free baseline approach, the constrained baseline approach led to similar true positive (power) rates but much higher false positive (Type I error) rates. The free baseline approach should be preferred when the referent indicator is unbiased. When the referent assumption is violated, the false positive rate was unacceptably high for both free and constrained baseline approaches, and the true positive rate was poor regardless of whether the free or constrained baseline approach was used. Neither the free or constrained baseline approach can be recommended when the referent indicator is biased. We recommend paying close attention to ensuring the referent indicator is unbiased in tests of cluster bias. All Mplus input and output files, R, and short Python scripts used to execute this simulation study are uploaded to an open access repository.

## Introduction

Measurement invariance can be demonstrated for a measurement instrument if the instrument functions equivalently, in a probabilistic sense, over subpopulations. In other words, measurement invariance exists if two individuals with equal standing on the construct being assessed, but sampled from different subpopulations, have the same expected test score. This has been explained by numerous methodologists now including Drasgow (1982, 1984), Mellenbergh (1989), Meredith (1993), Millsap (2012), and Vandenberg and Lance (2000). These authors have all shown that without demonstrating measurement invariance, conclusions about differences in latent means are dubious. More recent papers by Chen (2008) and Guenole and Brown (2014) have shown that, in addition, a lack of invariance leads to biased estimates of relationships between latent variables across groups, if the lack of invariance (or non-invariance) is not appropriately modeled.

The goal of this article is to present a Monte Carlo study that compares the effectiveness of two strategies for examining invariance simultaneously over many groups (i.e., cluster bias) in the context of multilevel confirmatory factor analysis (multilevel CFA). In the remainder of this article, we first present a brief literature review and theoretical framework for invariance testing in a multilevel CFA context. Following this overview, we describe the goals of the simulation study, the simulation conditions and rationale for their selection, and the simulation results. We then discuss the practical implications of this article for applied researchers in our discussion section.

## Literature review and theoretical framework

Methods for detecting items that violate measurement invariance are well developed. Tests for continuous indicator models are primarily based on confirmatory factor analysis (CFA). CFA tests involve first examining configural invariance, or whether the same number of latent dimensions are present in the data for each group. The configural invariance tests are followed by examining factor loadings of items across groups for equivalence. If the factor loadings are not equivalent across groups, the test items are said to violate metric or weak factorial invariance in a CFA framework.

If the factor loadings are equivalent, but intercepts are not equivalent, the items are said to violate scalar or strong invariance in CFA. Finally, equivalence of item error variances is also studied in CFA. If error variances are equal across groups, the items are said to show *strict invariance*. Chan (1998) has described the assumption of equivalent error variance as an unrealistic expectation in many applied situations. For a recent special issue on the topic of measurement invariance from the structural equation modeling perspective, see van de Schoot et al. (2015). When there are just two subpopulations of interest, say male and female in the context of CFA, measurement invariance is often examined using multiple indicator multiple causes models (Joreskog and Goldberger, 1975; Kim et al., 2012b; MIMIC: Chun et al., 2016), restricted factor analysis (Oort, 1998; RFA: Barendse et al., 2010), or multiple group models based on mean and covariance structures analysis (Sorbom, 1974; MACS: Byrne et al., 1989; Cheung and Rensvold, 1999). In these approaches the groups are fixed, and the method does not treat the groups across which invariance is examined to be a sample from a population.

Research attention has started to focus on situations where there are a large number of groups across which researchers wish to examine invariance. Examples might include invariance of a values questionnaire across cultures (e.g., Cheng et al., 2014; Cieciuch et al., 2017; Jang et al., 2017) or the invariance of a measurement instrument across classrooms in schools (e.g., Muthén, 1991). In cases like this, if there are a large number of groups, the usual multi-group approach can be cumbersome. Instead, measurement invariance can be examined with meta-analytic approaches (e.g., Cheung et al., 2006), recently developed fixed mode of variation approaches (i.e., approaches that do not attempt to make inferences beyond the groups in the analysis) like alignment optimization (Asparouhov and Muthén, 2014; Cieciuch et al., 2014), or multilevel confirmatory factor analysis (multilevel CFA: Muthén, 1994; Rabe-Hesketh et al., 2004). Multilevel CFA, the focus of this article, treats the grouping variable as a random mode of variation. In other words, it views the groups as a sample of groups from a larger population of groups (Muthén, 1994; Kim et al., 2012a; Jak et al., 2013; Ryu, 2014).

With two-level data, invariance can be examined at level-1 or level-2, but is more commonly studied at level-2, as described by Muthén et al. (1997), for example. Level-2 bias detection is the focus of the simulations to be presented in this article. Early papers by Muthén (1994) and Rabe-Hesketh et al. (2004) established the prerequisites for measurement invariance in multilevel measurement models, and a series of recent papers by Jak and her colleagues (Jak et al., 2013, 2014; Jak and Oort, 2015) further outlined the logic for tests of multilevel measurement invariance, or cluster bias. In two-level CFA the covariance matrix is decomposed as:

(1) $$\begin{array}{r} \Sigma_{\text{total}}=\Sigma_{\text{between}}+\Sigma_{\text{within}}. \end{array}$$

If there is no measurement bias at level-2 the following models will fit the data for *p* observed and *k* latent variables:

(2) $$\begin{array}{r} \Sigma_{\text{between}}=\Lambda \Phi_{\text{between}}\Lambda^{\prime } \end{array}$$

(3) $$\begin{array}{r} \Sigma_{\text{within}}=\Lambda \Phi_{\text{within}}\Lambda^{\prime }+\;\Theta_{\text{within}} \end{array}$$

where Φ_between_ and Φ_within_ are *k* × *k* latent variable covariance matrices, Λ is a *p* × *k* matrix of factor loadings, and Θ_within_ is a diagonal *p* × *p* matrix of residual variances. Cluster bias is related to the concept of isomorphism, which refers to equal factor loadings across levels. Isomorphism has important consequences for conclusions about the similarity of relationships between variables across levels, which in turn is referred to as homology (Tay et al., 2014; Guenole, 2016). However, absence of cluster bias is a stronger assumption than isomorphism, because cluster bias refers to non-zero residual variance at level-2, in a model where isomorphism holds.

In practice, the Jak et al. (2013) procedure for testing multilevel invariance unfolds as follows. First, *configural invariance* is examined. The configural invariance model holds where the pattern of factor loading coefficients is consistent across the within and between levels of the multilevel CFA model. Next, the *cluster invariance* model is fit to the data where factor loadings and intercepts are equivalent across clusters. The data support a *cluster invariance* or strong invariance model when the factor loadings are equivalent across levels and level-2 item residual variances are not significantly different from zero. Jak and Oort (2015) noted that the result of bias in factor loadings and intercepts manifests as level-2 residual variance when factor loadings are constrained across levels, and the test for cluster bias does not differentiate the source for the bias (i.e., whether it is intercept or factor loading non-invariance across level-2 clusters), rather, it simply tests for the presence of measurement bias. If the level-2 residual variances are significantly different from zero, the bias could be in factor loadings and/or intercepts. In this article, we focus attention on uniform bias which we simulate by incorporating the direct effect of a level-2 violator, described more in the methodology section.

In the sections that follow, we contrast free and constrained baseline approaches to testing measurement bias. Importantly, the free vs. constrained distinction in the context of item level testing is distinct from scale level testing for cluster bias where the configural model is contrasted with the scalar invariance model. The item level procedures we investigate here are likely to be followed by researchers if they find that cluster invariance is violated.

### Constrained baseline approaches to measurement invariance testing

An item level approach to cluster bias based on Jak et al.'s (2013) procedure can be considered a constrained (of “fixed”) baseline approach. This is because it begins by fixing all parameters to be tested to be either equal across levels in the case of factor loadings or zero in the case of between level residual variances. In this approach, the overall fit of the fully constrained model is first evaluated. The alternative model then frees the level-2 residual variances for the studied item(s). An evaluation of the improvement in model fit from freeing the item parameters is then made, using a test such as the likelihood ratio difference test or one of its variations. Statistical significance indicates that the model with constraints fits significantly worse than the model without constraints, and the item exhibits measurement bias. Conversely, statistical non-significance indicates that the model with constraints does not fit significantly worse than the model without constraints, and the item does not exhibit measurement bias. Stark et al. (2006) noted that constrained baseline procedures are the typical approach used by researchers coming from an item response modeling tradition.

### Free baseline approaches to measurement invariance testing

An alternative approach to the constrained baseline strategy begins with a free baseline where minimal constraints are imposed. In this approach, the minimally identifiable model is estimated as the baseline model. The alternative model then constrains the parameters of the items being tested across groups (one factor loading and one residual variance), but leaves parameters for all other items free across groups. An evaluation of the difference in fit between the constrained and unconstrained models is then made using a test such as the likelihood ratio test. Statistical non-significance indicates that fixing the item parameters equal across groups does not yield a statistically significant decrement in model fit, and that the item does not exhibit bias. Statistical significance indicates that the item does exhibit measurement bias across groups. Stark et al. (2006) noted that free baseline procedures are the typical approach used by researchers coming from a structural equation modeling tradition.

### Competing rationales for constrained and free baseline approaches

Readers should note that while the free vs. constrained baseline issue has not been examined in the context of multilevel measurement invariance, numerous related examples exist in the traditional two-group case under the label of *iterative* bias detection. These include applied instances (e.g., Navas-Ara and Gómez-Benito, 2002) as well as Monte Carlo studies (e.g., Oort, 1998; Barendse et al., 2012). In the iterative approach, results of previous item tests are incorporated into the baseline model for testing of subsequent items. In the method adopted in this article, we always revert to the original free or constrained baseline for tests of subsequent items.

Researchers have offered different rationales for examining invariance with free and constrained baseline approaches. Constrained baseline approaches might be a reasonable approach if the majority of items are believed to be invariant, perhaps based on past research. Furthermore, Jak and Oort (2015) showed with simulations that a constrained baseline approach leads to reasonably accurate conclusions under some circumstances when examining cluster bias. A constrained baseline might also be defended on the basis that more stable parameter estimates are achieved when the linking required to establish a common metric is based on more than one item (Stark et al., 2006).

Nevertheless, there may be an impact on subsequent tests of model fit if the unrestricted model is misspecified. To calculate the log-likelihood ratio test two models are estimated, an unrestricted model M_0_, and a restricted model, M_1_. The log-likelihood statistic is calculated by comparing the log-likelihoods of the two models: LRT = −2 x (ℓ_0_ − ℓ_1_). The LRT statistic is distributed as χ^2^ with degrees of freedom equal to the difference in the number of estimated parameters between the models, but this is only so if the unrestricted model is correctly specified. If it is not, it could see a reduction in statistical power to detect bias when it exists, and increased Type I errors where non-biased items are identified as biased. From a logical perspective, the cautious and strongest theoretical approach seems to be to use the free baseline procedure. Indeed, simulation studies in the two-group context have revealed greater accuracy for the free baseline approach across dominance and unfolding item response models (Stark et al., 2006; Wang et al., 2013; Chun et al., 2016). Comparing the free and constrained baseline approaches with Monte Carlo methods in a multi-level CFA context is the central goal of this article. Importantly, while the general procedures of free and constrained approaches to invariance testing are not new, the two procedures we examine have never been evaluated before in the context of testing cluster bias. There are also other possible approaches to free and constrained baseline testing that this article does not address. We return to these alternative approaches in our discussion.

We broadly follow the recommendations of Paxton et al. (2001). Our hypotheses were as follows:

*H1*: A free baseline approach will provide greater decision accuracy in terms of true positive rates and false positive rates in comparison to the constrained baseline approach.
*H2*: The improved performance of the free baseline approach will be even more observable in terms of true positive rates and false positive rates with factors expected to increase decision accuracy (i.e., higher ICC, larger L1 and L2 sample sizes, more non-invariant items, and higher magnitude bias).

We were interested in the performance of free and constrained baseline approaches where the referent item was free of bias and when it was biased, since in practice this important assumption may be easily violated. We did not have a hypothesis for the biased referent indicator section in our design and therefore treat this part of the analysis as exploratory.

## Method

### Design features

The simulation conditions for the most part follow the set up described by Jak et al. (2014), which provide a strong basis on which to evaluate the performance of cluster bias detection in multilevel CFA. In addition, we verified the appropriateness of these conditions by a review of existing simulation studies addressing multilevel CFA questions. In the sections that follow, we describe the simulation set up for the current study.

### Fixed features of simulation design

*Test length* was set at five items. Five items have been commonly used in measurement model simulation studies, and this number of items is very common in survey research where there is not sufficient room for longer scales.

*Continuous indicators* were simulated. Continuous item indicator factor models are common in survey work where research shows that so long as the number of scale points in a Likert scale model is greater than five, continuous factor models perform well.

*Replications* were set at 500 replications per cell which is consistent with the number of replications used in past studies and is expected to be a sufficient number of replications to achieve reliable results.

*Missing data* patterns were not included in our simulation, and so the impact of missing data patterns in multilevel measurement invariance falls outside the scope of our simulation.

*Level-2 violators* were simulated to introduce non-invariance in our simulations. The effect of the level-2 violator is to increase the variance for the biased item. We subsequently examined items for bias with tests of cluster bias.

### Experimental conditions

#### Level 1 samples sizes (three levels)

Level-1 sample sizes (L1N) were set at 2, 5, and 25, mirroring the simulation conditions presented since cluster samples sizes of two are common in dyad research, five are common in small group research, and 25 is common in educational and organizational research.

#### Level 2 sample sizes (two levels)

Level-2 sample sizes (L2N) were set at 50 or 100. These cluster sizes can be considered moderate and large, and were chosen because results of simulations in multilevel CFA contexts by Maas and Hox (2005) show sample sizes in this range are required for estimation accuracy.

#### Intraclass correlations (ICCs) (three levels)

Based on a review of ICCs in simulation studies including Maas and Hox (2005), the ICCs were set 0.10, 0.20, and 0.30. While smaller ICCs have been investigated by some researchers (e.g., Depaoli and Clifton examined ICCs of 0.02), larger ICCs are both common in applied settings and less likely to result in inadmissible solutions.

#### Number of biased items (three levels)

We included conditions with 0, 1, and 2 biased items. This allowed examining the impact of the severity of baseline misspecification on decision accuracy. The no bias condition was included simply as a simulation baseline to check the basal Type I error, following Kim et al. (2012b).

#### Size of bias (three levels)

We incorporated three levels of bias: no bias, small bias, and large bias. We set the bias to be one and five percent of the total variance of the indicator for small and large bias respectively. We did this using the methodology of increasing the variance of the biased item by allowing a direct effect of a level-2 violator variable, described below under data generation. This approach has been used in past simulation studies, including Barendse et al. (2012). Again, the no bias condition was included only as a simulation baseline check.

#### Biased or unbiased anchor item (two levels)

We examined the performance of the free and constrained baseline models when the anchor item was biased and when it was unbiased. Note that for levels of bias with 2 biased items under the biased anchor item this meant there were, in fact, 3 biased items: 2 biased items with a biased anchor item. The size of the bias in the anchor item was set to match the size of the item bias in the remaining item(s), i.e., 1 or 5% bias.

#### Summary of factorial design

Our design included the baseline check conditions of three L1N × two L2N × three intraclass correlation levels x one combination of biased items and levels of bias (i.e., zero bias) = 18 conditions; along with three L1N × two L2N × three intraclass correlation levels x four combinations of bias items and levels of bias (i.e., one or two bias items with either small, or large bias) x two anchor item levels (biased and unbiased) = 144 conditions. Each condition was analyzed using two detection strategies (i.e., both the free and the constrained baseline strategies).

### Data generation

**Figure 1:**
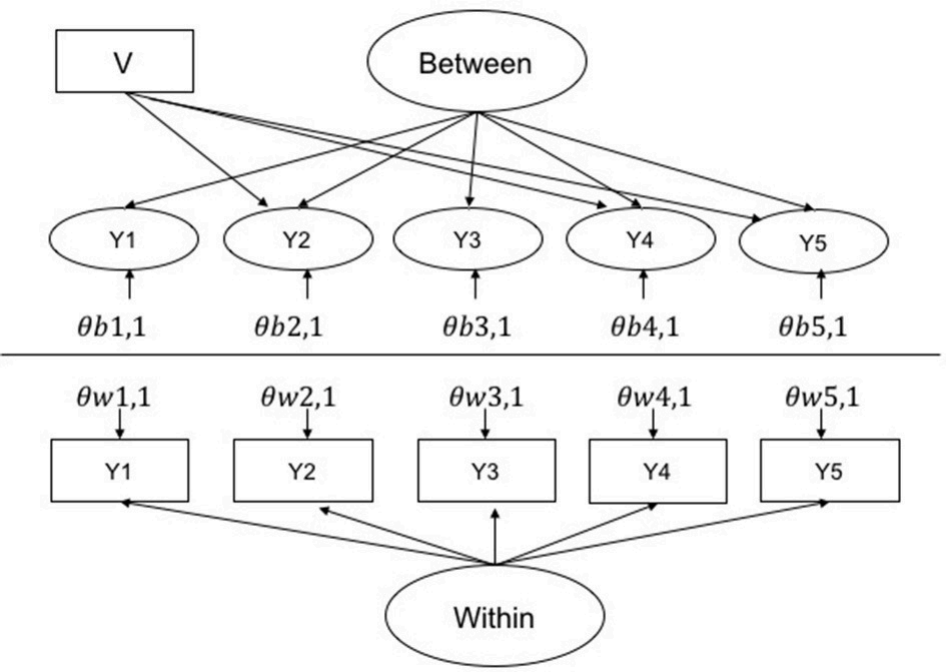
Multilevel CFA base model for the most biased condition (biased referent 2, and two further biased items 4 and 5). V is a level 2 violator that causes bias in the affected indicators by increasing there variance by either 1 and 5%. The variance of the within factor was set at 1 and the between was varied to adjust the ICC. Loading on within and between were set at 1 for all items. θs are errors where w and b represent within and between, respectively, and 1, 1 indicates the first indicator of the factor, etc.

All simulated data were generated as continuous multivariate normal using Mplus version 8 (Muthén and Muthén, 1998-2017). All input scripts, outputs scripts, and python scripts used to extract model summary statistics are available at the following figshare link: https://figshare.com/s/23427e33be46d406b5d0. The base model from which all simulation models can be derived is presented in Figure 1. In the unbiased referent indicator conditions, bias was simulated on items 2 and 4. In the biased referent indicator conditions, bias was simulated on items 2, 4, and 5.

### Model identification

#### Unbiased anchor

To identify the metric of the latent factors in the free baseline approach we fixed the first factor loading of each factor on within and between levels at one. This item was not biased. The baseline model in the constrained approach was identified by fixing the factor loading of the first indicator of each item at 1 and having all remaining within and between level factor loadings equal and all level two residual variances set at zero—thus violating the assumption of an unbiased referent indicator.

#### Biased anchor

To identify the metric of the factors in the free baseline approach we fixed the second factor loading of each factor on within and between levels at one. This item was a biased item. All remaining factor loadings and residual variances were freely estimated. To identify the metric of the latent factors in the constrained baseline condition, we again fixed the within and between level factor loadings of item 2, which was biased, at 1. All remaining item factor loadings were constrained equal across levels and all level-2 residual variances were fixed at 0.

### Estimation

We estimated all models using robust maximum likelihood estimation (MLR).

### Testing for cluster bias from constrained and unconstrained baselines

To test the invariance of items in the free baseline condition, we examined the significance of the difference in −2 × the log-likelihood of the restricted model and the unrestricted model. In the restricted model the within and between level factor loadings were equal and the residual variances for the tested item was zero, respectively. In the unrestricted model the factor loadings and residual variance for the tested item were free. This yields a test statistic that is χ^2^ distributed with 2 degrees of freedom. To test for bias of items under the constrained baseline, the starting model was the reduced model where all factor loadings were fixed equal and level-2 residual variances were zero; the unrestricted model freed the within and between level factor loadings for the tested item along with its between level residual variances. The test statistic was compared against a χ^2^ distribution with two degrees of freedom.

This restricted baseline approach differs from the Jak et al. (2014) procedure in that their approach evaluates the improvement in fit from moving from the model with constrained factor loadings for an item with its residual variance at zero to the same model but with only the residual variance freed. However, for comparability with the free baseline approach outlined, here we examine the improvement in fit that results from freeing both the item residual variance and the across level factor loading constraint simultaneously.

A correction is sometimes applied to calculate the differences in χ^2^ between nested models because differences in −2 × log-likelihood values are not χ^2^ distributed under the maximum likelihood estimator with robust standard errors (Satorra and Bentler, 2001). However, Jak and Oort (2015, p. 440), citing Cham et al. (2012) recommended using the uncorrected difference in −2 × log-likelihood between the nested models because in the context of difference testing a procedure with the correction does not perform better than an approach without the correction. They reported that their log-likelihood differences were sometimes negative, and they considered these non-significant. A strictly positive χ^2^ difference test has been developed for the situations where the negative values occur, and it has been suggested as potentially being relevant in other cases such as small samples. However, recommendations to date are unclear with regard to whether this strictly positive variation ought to be applied in all cases. For this reason, we used unscaled −2 × log-likelihood difference tests.

### True positive rates and false negative rates

True positives rates were calculated as the proportion of simulation runs within each condition where biased items were correctly identified as biased with the two degree of freedom log-likelihood difference test. False positive rates were calculated as the proportion of simulation runs within each condition where unbiased items were incorrectly identified as exhibiting bias with the two degree of freedom log-likelihood difference test. Power corresponds to the true positive rate, while Type I error corresponds to the false positive rate.

When estimating many multilevel models with small variances and low L1N, numerous estimation challenges are likely to emerge, particularly in these models. These include runs where (a) the software does not complete a replication, (b) the software makes estimation adjustments due to the estimation hitting saddle points, (c) models converge to inadmissible solutions, and (d) negative log-likelihood difference values result. In instances when either model required for a log-likelihood difference did not converge, the result was not counted as a true positive or a false positive, but the proportion of true positives and false positives observed for the cell is still expressed as a proportion of the 500 intended runs.

We observe differences in reporting practices with regard to whether runs with adjustments due to saddle points and inadmissible solutions are summarized over or omitted in Monte Carlo results for measurement invariance. In this study, if the software converged to an inadmissible solution, and when an adjustment was made due to hitting a saddle point, the log-likelihood difference test was still conducted and summarized in the same way as for log-likelihood difference tests for admissible solutions. In addition, as with the study reported by Jak et al. (2013), on numerous occasions the log-likelihood difference was negative. In the Jak et al. (2013) study, the authors counted these to be non-significant differences, however, in the present study we considered these to be inconclusive and did not count them in our analyses as constituting a true positive or a false positive occurrence.

## Results

### Simulation baseline check

Under the constrained baseline approach with no biased items the false positive rate (based on testing item 2 for bias) and the true positive rate (based on testing item 3 for bias) are both false positive rates, because there is no bias. The detection rate should be around the nominal significance level of 0.05 across all experimental conditions, because the baseline model and the comparison models are always correctly specified. Table 1 reveals that the false positive rate based on testing item 2 for bias in the constrained baseline condition was always slightly lower than 0.05. The false positive rate based on testing item 3 for bias was also slightly lower than 0.05 across all conditions. Similarly, under the free baseline approach, the false positive rate (based on testing item 2 for bias) and the true positive rate (based on testing item 3 for bias) both constitute false positive rates. These are expected to be around 0.05 across all experimental conditions. Table 1 shows that the false positive rate based on testing item 2 for bias was always slightly lower than 0.05, and the false positive rate based on testing item 3 for bias was also always slightly lower than the expected 0.05.

**Table 1:** True positive rates and false positive rates for unbiased anchor with no bias.

						**Free baseline**				**Fixed baseline**			
**Cell**	**L2N**	**L1N**	**ICC**	**Items**	**Size (%)**	**NLD**	**FP-2**	**NLD**	**FP-3**	**NLD**	**FP-2**	**NLD**	**FP-3**
1	50	2	0.10	0	0	21	0.03	13	0.02	225	0.02	229	0.02
31	50	2	0.20	0	0	20	0.02	18	0.03	243	0.02	226	0.02
61	50	2	0.30	0	0	17	0.03	10	0.03	232	0.02	237	0.02
6	50	5	0.10	0	0	49	0.02	21	0.02	253	0.01	239	0.01
36	50	5	0.20	0	0	20	0.03	31	0.02	193	0.02	213	0.03
66	50	5	0.30	0	0	24	0.01	27	0.02	211	0.01	215	0.02
11	50	25	0.10	0	0	38	0.02	34	0.02	130	0.01	121	0.03
41	50	25	0.20	0	0	33	0.03	28	0.01	105	0.04	122	0.01
71	50	25	0.30	0	0	32	0.03	23	0.02	109	0.03	104	0.01
16	100	2	0.10	0	0	26	0.03	26	0.03	277	0.01	268	0.02
46	100	2	0.20	0	0	37	0.04	19	0.03	248	0.02	258	0.02
76	100	2	0.30	0	0	36	0.03	41	0.02	215	0.02	241	0.01
21	100	5	0.10	0	0	20	0.03	29	0.04	186	0.02	208	0.03
51	100	5	0.20	0	0	22	0.02	24	0.02	202	0.02	172	0.02
81	100	5	0.30	0	0	22	0.02	34	0.03	214	0.02	203	0.03
26	100	25	0.10	0	0	31	0.02	39	0.02	107	0.01	126	0.02
56	100	25	0.20	0	0	32	0.02	34	0.03	108	0.03	127	0.03
86	100	25	0.30	0	0	26	0.02	32	0.03	102	0.02	123	0.02

*L2N, level-2 sample size; L1N, level −1 sample size; ICC, intraclass correlation; Items, number of biased items; NLD, count of negative log-likelihood difference test results; FP-2, false positive rate for item 2; FP-3, false positive rate for item 3*.

### Negative log-likelihood difference tests

In the simulation baseline conditions, and in all conditions that follow, the occurrences of negative log-likelihood difference tests were substantially higher under the constrained baseline detection strategy, and precise frequencies can be observed in Table 2 through **Table 5**.

**Table 2:** True positive and false positive rates for unbiased anchor with one biased item.

						**Free baseline**				**Fixed baseline**			
**Cell**	**L2N**	**L1N**	**ICC**	**Items**	**Size (%)**	**NLD**	**TP**	**NLD**	**FP**	**NLD**	**TP**	**NLD**	**FP**
2	50	2	0.10	1	1	12	0.04	19	0.04	207	0.02	200	0.03
3	50	2	0.10	1	5	4	0.14	21	0.06	216	0.01	216	0.01
32	50	2	0.20	1	1	25	0.05	15	0.05	198	0.02	220	0.02
33	50	2	0.20	1	5	7	0.09	20	0.04	84	0.07	253	0.02
62	50	2	0.30	1	1	18	0.06	17	0.05	208	0.04	221	0.02
63	50	2	0.30	1	5	2	0.13	27	0.04	75	0.12	240	0.02
7	50	5	0.10	1	1	11	0.07	20	0.02	137	0.07	225	0.02
8	50	5	0.10	1	5	1	0.47	16	0.04	11	0.51	169	0.04
37	50	5	0.20	1	1	13	0.06	20	0.02	130	0.07	188	0.02
38	50	5	0.20	1	5	1	0.59	21	0.02	5	0.60	193	0.02
67	50	5	0.30	1	1	12	0.06	36	0.02	98	0.08	189	0.03
68	50	5	0.30	1	5	0	0.66	15	0.02	3	0.69	198	0.03
12	50	25	0.10	1	1	2	0.57	25	0.01	4	0.60	99	0.03
13	50	25	0.10	1	5	0	1.00	16	0.02	0	1.00	56	0.07
42	50	25	0.20	1	1	0	0.68	21	0.02	4	0.71	84	0.02
43	50	25	0.20	1	5	0	1.00	21	0.02	0	1.00	59	0.08
72	50	25	0.30	1	1	1	0.75	33	0.02	1	0.78	111	0.03
73	50	25	0.30	1	5	0	1.00	26	0.02	0	1.00	59	0.10
17	100	2	0.10	1	1	21	0.07	27	0.06	203	0.02	244	0.01
18	100	2	0.10	1	5	10	0.18	28	0.04	72	0.16	254	0.02
47	100	2	0.20	1	1	18	0.03	31	0.04	190	0.03	245	0.03
48	100	2	0.20	1	5	5	0.20	30	0.04	44	0.18	227	0.02
77	100	2	0.30	1	1	17	0.03	20	0.04	209	0.02	212	0.03
78	100	2	0.30	1	5	8	0.23	39	0.01	47	0.24	235	0.02
22	100	5	0.10	1	1	15	0.09	24	0.03	102	0.10	170	0.01
23	100	5	0.10	1	5	0	0.84	27	0.03	0	0.87	175	0.04
52	100	5	0.20	1	1	7	0.11	23	0.02	64	0.10	186	0.02
53	100	5	0.20	1	5	0	0.89	25	0.03	0	0.92	170	0.03
82	100	5	0.30	1	1	8	0.10	20	0.03	67	0.10	197	0.02
83	100	5	0.30	1	5	0	0.94	30	0.04	0	0.96	146	0.04
27	100	25	0.10	1	1	1	0.87	28	0.04	1	0.89	91	0.04
28	100	25	0.10	1	5	0	1.00	31	0.02	0	1.00	36	0.15
57	100	25	0.20	1	1	0	0.94	29	0.02	0	0.95	97	0.05
58	100	25	0.20	1	5	0	1.00	23	0.02	0	1.00	40	0.18
87	100	25	0.30	1	1	0	0.98	30	0.03	0	0.99	86	0.04
88	100	25	0.30	1	5	0	1.00	29	0.02	0	1.00	37	0.18

*L2N, level-2 sample size; L1N, level −1 sample size; ICC, intraclass correlation; Items, number of biased items; NLD, count of negative log-likelihood difference test results; TP, true positive rate; FP, false positive rate*.

### Unbiased referent item results

#### Summary of true positive and false positive rates

True positive (power) and false positive (Type I error) rates are summarized in Tables 2, 3. For the unbiased anchor conditions, the overall true positive rate for the free baseline approach was 0.44, while the overall true positive rate for the constrained baseline approach was similar at 0.42. The overall false positive rate for the free baseline approach was 0.04, while the overall false positive rate for the constrained baseline was unacceptably high at 0.14. This is because in contrast to the baseline check condition where the baseline model was always correctly specified and the fixed baseline approach performed well in terms of false positives, in these conditions the baseline model was always misspecified and the false positives are too high. We now further explore factors associated with variability in these true positive and false positive rates with ANOVA models.

**Table 3:** True positive and false positive rates for unbiased anchor with two biased items.

						**Free baseline**				**Fixed baseline**			
**Cell**	**L2N**	**L1N**	**ICC**	**Items**	**Size (%)**	**NLD**	**FP**	**NLD**	**FP**	**NLD**	**FP**	**NLD**	**FP**
4	50	2	0.10	2	1	12	0.05	13	0.03	206	0.03	239	0.01
5	50	2	0.10	2	5	5	0.16	21	0.04	186	0.06	193	0.02
34	50	2	0.20	2	1	25	0.05	14	0.04	235	0.03	227	0.02
35	50	2	0.20	2	5	10	0.19	14	0.04	157	0.08	181	0.03
64	50	2	0.30	2	1	13	0.05	20	0.03	197	0.04	193	0.03
65	50	2	0.30	2	5	8	0.18	13	0.06	115	0.06	174	0.04
9	50	5	0.10	2	1	14	0.05	21	0.03	159	0.03	194	0.02
10	50	5	0.10	2	5	4	0.46	17	0.02	42	0.23	113	0.07
39	50	5	0.20	2	1	11	0.05	19	0.02	158	0.03	184	0.03
40	50	5	0.20	2	5	5	0.39	13	0.03	34	0.30	85	0.07
69	50	5	0.30	2	1	16	0.07	18	0.03	138	0.05	176	0.03
70	50	5	0.30	2	5	3	0.39	19	0.03	20	0.32	104	0.08
14	50	25	0.10	2	1	7	0.27	17	0.03	20	0.28	65	0.07
15	50	25	0.10	2	5	0	1.00	18	0.09	0	1.00	1	0.81
44	50	25	0.20	2	1	5	0.33	15	0.02	15	0.32	61	0.08
45	50	25	0.20	2	5	0	1.00	15	0.04	0	1.00	1	0.85
74	50	25	0.30	2	1	1	0.39	15	0.05	7	0.40	47	0.13
75	50	25	0.30	2	5	0	1.00	14	0.02	0	1.00	0	0.89
19	100	2	0.10	2	1	26	0.05	23	0.04	215	0.02	224	0.02
20	100	2	0.10	2	5	11	0.27	30	0.04	133	0.07	203	0.03
49	100	2	0.20	2	1	20	0.05	21	0.04	204	0.03	233	0.02
50	100	2	0.20	2	5	13	0.28	31	0.07	121	0.09	187	0.06
79	100	2	0.30	2	1	28	0.05	22	0.03	206	0.05	236	0.03
80	100	2	0.30	2	5	9	0.24	36	0.05	91	0.11	196	0.04
24	100	5	0.10	2	1	17	0.07	34	0.03	2	0.87	1	0.86
25	100	5	0.10	2	5	0	0.79	21	0.03	7	0.53	72	0.13
54	100	5	0.20	2	1	19	0.05	29	0.03	126	0.06	159	0.04
55	100	5	0.20	2	5	1	0.65	23	0.05	5	0.54	64	0.16
84	100	5	0.30	2	1	77	0.83	31	0.03	118	0.09	160	0.05
85	100	5	0.30	2	5	0	0.72	15	0.09	0	0.69	44	0.20
29	100	25	0.10	2	1	1	0.47	12	0.06	7	0.49	29	0.14
30	100	25	0.10	2	5	0	1.00	17	0.08	0	1.00	0	1.00
59	100	25	0.20	2	1	1	0.62	18	0.06	4	0.64	36	0.18
60	100	25	0.20	2	5	0	1.00	13	0.04	0	1.00	0	0.99
89	100	25	0.30	2	1	0	0.71	14	0.06	0	0.70	20	0.23
90	100	25	0.30	2	5	0	1.00	19	0.05	0	1.00	0	0.99

*L2N, level-2 sample size; L1N, level −1 sample size; ICC, intraclass correlation; Items, number of biased items; NLD, count of negative log-likelihood difference test results; TP, true positive rate; FP, false positive rate*.

#### True positive rates (power)

In an ANOVA model for the unbiased anchor condition where the true positive rate was predicted by all independent variables the significant independent variables at *p* < 0.05 were Level-2 N ($\eta_{\text{L}2\text{N}}^{2}$ = 0.031), Level-1 N ($\eta_{\text{L}1\text{N}}^{2}$ = 0.566), the number of biased items ($\eta_{\text{no}.\text{ biased items}}^{2}$ = 0.011) and the size of the bias ($\eta_{\text{size bias}}^{2}$ = 0.175). Non-significant effects included ICC ($\eta_{\text{ICC}}^{2}$ = 0.003), and free vs. fixed ($\eta_{\text{free v fixed}}^{2}$ = 0.001, where the free baseline was coded 0 and the constrained baseline was coded 1). It is important not to over-interpret small but statistically significant effects, so here we consider effect sizes in relation to Cohen (1988) criteria. The only effects that met Cohen's (1988) criterion for being at least a moderate effect (0.058) were Level-1 N and the size of bias. Next, an examination of all two-way interactions indicated that the interaction between the ICC and the number of biased items was significant at *p* < 0.05 ($\eta_{\text{ICC }\times \text{ no}.\text{ biased items}}^{2}$ = 0.008), although by Cohen's benchmark this effect is small. There were no interactions involving the free vs. fixed baseline variable manipulation.

#### False positive (Type I error) rates

In an ANOVA model for the unbiased anchor condition where the false positive rate was predicted by all independent variables the significant independent variables at *p* <0.05 were L1N ($\eta_{\text{L}1\text{N}}^{2}$ = 0.097), number of biased items ($\eta_{\text{no}.\text{ biased items}}^{2}$ = 0.071), size of bias ($\eta_{\text{size bias}}^{2}$ = 0.036), and whether a free or fixed baseline was used ($\eta_{\text{free v fixed}}^{2}$ = 0.072). These effects are all moderate by Cohen's (1988) criteria, aside from the effect of the size of the bias, which was small. We next explored all two-way interactions. This indicated that the following interactions were significant at *p* < 0.05: L1N × number of biased items ($\eta_{\text{L}1\text{N }\times \text{ no. biased items}}^{2}$ = 0.067), L1N × size of bias ($\eta_{\text{L}1\text{N }\times \text{ size bias}}^{2}$ = 0.072), L1N × free vs. fixed baseline ($\eta_{\text{L}1\text{N }\times \text{ free v fixed}}^{2}$ = 0.100), number of biased items × size of bias ($\eta_{\text{no}.\text{ biased items }\times \;size\;bias}^{2}$ = 0.022), number of biased items × free vs. fixed baseline ($\eta_{\text{no}.\text{ biased items }\times \text{ free v fixed}}^{2}$ = 0.055), and size of bias × free vs. fixed baseline ($\eta_{\text{size bias }\times \text{ free v fixed}}^{2}$ = 0.032). Interaction effect sizes that were at least moderate by Cohen's standard, therefore, include L1N × number of biased items, L1N × size of bias, L1N × free vs. fixed baseline ($\eta_{\text{L}1\text{N }\times \text{ free v fixed}}^{2}$ = 0.100). The interaction for the number of biased items × free vs. fixed baseline was also very close to being a moderate effect size.

**Figure 2:**
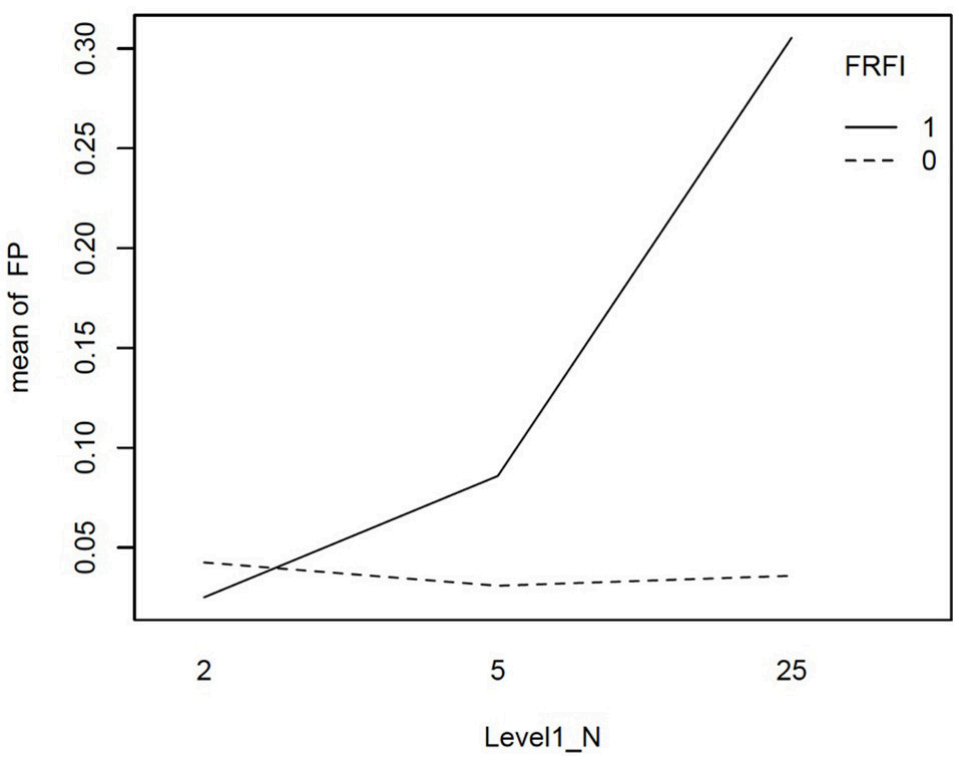
Interaction plot for the effect of level-1 sample size and the free vs. fixed baseline choice on the false positive rate when the referent indicator is unbiased. FRFI, free vs. fixed, where 0 is free and 1 is fixed; FP, false positive rate; L1N, level 1 sample size; where 2, 5, and 25 are the level 1 sample sizes studied.

**Figure 3:**
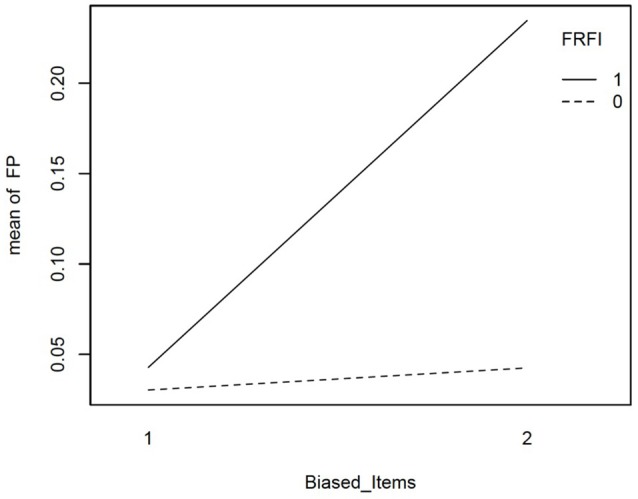
Interaction plot for the effect of number of biased items and the free vs. fixed baseline choice on the false positive rate when the referent indicator is unbiased. FRFI, free vs. fixed, where 0 is free and 1 is fixed; FP, false positive rate; Biased items, number of biased items where 1 = 1 biased item and 2 = 2 biased items.

We explored the interactions involving the free vs. fixed baseline manipulation, our focal independent variable, further with graphical plots. Figure 2 depicts the interaction between L1N and free vs. fixed baseline on the false positive rate. This figure reveals that moving from the lowest L1N size to the highest L1N size for the free baseline model results in no notable change in the false positive rate. On the other hand, moving from the lowest L1N size to the highest L1N size with a fixed baseline leads to a substantial jump in the false positive rate. Figure 3 depicts the interaction between the number of biased items and the free vs. fixed baseline strategy. This figure reveals that with 1 biased item present, the free and fixed baseline approaches perform similarly in terms of controlling the Type I error rate.

**Figure 4:**
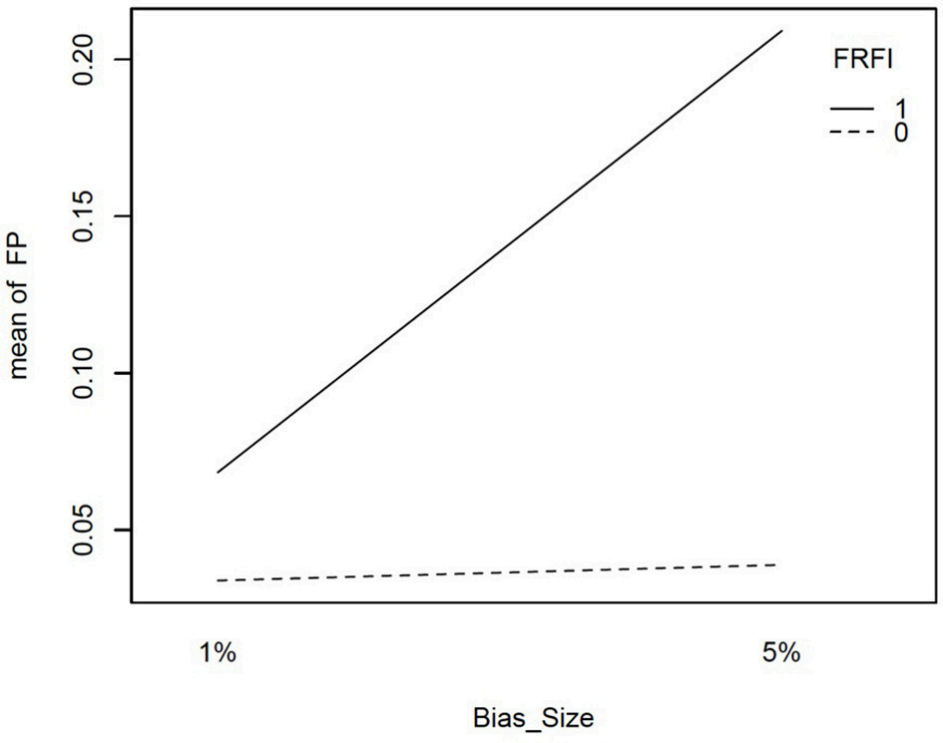
Interaction plot for size if item bias and free vs. fixed baseline approach on the false positive rate when the referent indicator is unbiased. FRFI, free vs. fixed, where 0 is free and 1 is fixed; FP, false positive rate; Bias size, size of the bias, where 1% = 1% bias and 5% = 5% bias.

However, in the presence of two biased items, the free baseline approach performs considerably better at controlling the Type I error rate. The final interaction involving our focal independent variable was for free vs. fixed and the size of the bias. This interaction is plotted in Figure 4. It reveals that when the size of the bias is small, the free and fixed baseline approaches perform similarly, albeit with a lower Type I error rate for the free baseline approach. When the size of the bias is large, the free baseline approach continues to control the Type I error rate appropriately. The Type I error rate for the fixed baseline approach, however, rises to an unacceptable level.

### Biased referent item results

#### Summary of true positive and false positive rates

True positive (power) and false positive (Type I error) rates for the biased referent indicator are summarized in Tables 4, 5. These tables indicate that the false positive rate is poorly controlled when the anchor item is biased, regardless of whether a free baseline or a fixed baseline approach is adopted. The overall false positive rates under the free and fixed baseline approach were both unacceptable when the anchor item was biased, at 0.25 and 0.29 respectively. In this biased anchor condition, the overall true positive rate under the free baseline approach was 0.16, while it was higher at 0.28 under the fixed baseline approach. Comparison of the relative advantages of the free and fixed baseline across conditions is not meaningful given the unacceptably high false positive rates and poor power across both approaches. We do not explore this issue further here, instead we return to the topic of identifying an unbiased item in the discussion section below.

**Table 4:** True positive and false positive rates for biased anchor with one additional biased item.

						**Free baseline**				**Fixed baseline**			
**Cell**	**L2N**	**L1N**	**ICC**	**Items**	**Size (%)**	**NLD**	**TP**	**NLD**	**FP**	**NLD**	**TP**	**NLD**	**FP**
91	50	2	0.10	1	1	9	0.03	8	0.04	206	0.03	217	0.01
92	50	2	0.10	1	5	6	0.07	9	0.10	160	0.07	203	0.03
103	50	2	0.20	1	1	19	0.05	20	0.03	202	0.00	233	0.00
104	50	2	0.20	1	5	7	0.10	7	0.15	164	0.07	215	0.04
115	50	2	0.30	1	1	18	0.06	24	0.04	201	0.03	228	0.03
116	50	2	0.30	1	5	17	0.13	11	0.15	139	0.07	166	0.06
93	50	5	0.10	1	1	20	0.03	21	0.04	175	0.03	195	0.03
94	50	5	0.10	1	5	17	0.03	6	0.37	50	0.26	106	0.06
105	50	5	0.20	1	1	19	0.03	18	0.03	151	0.05	158	0.02
106	50	5	0.20	1	5	13	0.12	13	0.24	32	0.28	116	0.11
117	50	5	0.30	1	1	25	0.03	17	0.05	147	0.04	183	0.03
118	50	5	0.30	1	5	5	0.20	14	0.15	14	0.37	93	0.11
95	50	25	0.10	1	1	12	0.17	8	0.09	15	0.31	43	0.12
96	50	25	0.10	1	5	61	0.62	7	0.20	0	1.00	0	0.81
107	50	25	0.20	1	1	1	0.28	7	0.07	13	0.35	47	0.10
108	50	25	0.20	1	5	0	0.97	11	0.09	0	1.00	0	0.88
119	50	25	0.30	1	1	3	0.33	14	0.06	5	0.38	41	0.11
120	50	25	0.30	1	5	0	1.00	13	0.07	0	1.00	0	0.89
97	100	2	0.10	1	1	20	0.04	25	0.05	239	0.01	217	0.01
98	100	2	0.10	1	5	11	0.10	11	0.19	137	0.07	227	0.02
109	100	2	0.20	1	1	24	0.04	18	0.07	243	0.03	216	0.03
110	100	2	0.20	1	5	12	0.16	8	0.22	115	0.10	168	0.03
121	100	2	0.30	1	1	34	0.04	30	0.04	196	0.04	229	0.03
122	100	2	0.30	1	5	15	0.18	12	0.21	78	0.12	162	0.06
99	100	5	0.10	1	1	28	0.03	24	0.04	150	0.04	184	0.03
100	100	5	0.10	1	5	17	0.03	2	0.56	14	0.47	51	0.14
111	100	5	0.20	1	1	24	0.02	20	0.04	129	0.06	140	0.03
112	100	5	0.20	1	5	8	0.26	13	0.27	7	0.56	49	0.17
123	100	5	0.30	1	1	20	0.06	26	0.05	94	0.08	141	0.04
124	100	5	0.30	1	5	2	0.43	11	0.14	2	0.65	54	0.19
101	100	25	0.10	1	1	3	0.41	12	0.10	4	0.52	40	0.18
102	100	25	0.10	1	5	48	0.76	10	0.21	0	1.00	0	0.99
113	100	25	0.20	1	1	2	0.55	12	0.08	1	0.61	26	0.17
114	100	25	0.20	1	5	0	1.00	4	0.10	0	1.00	0	0.99
125	100	25	0.30	1	1	1	0.68	18	0.06	1	0.73	29	0.18
126	100	25	0.30	1	5	0	1.00	16	0.07	0	1.00	0	0.99

*L2N, level-2 sample size; L1N, level −1 sample size; ICC, intraclass correlation; Items, number of biased items; NLD, count of negative log-likelihood difference test results; TP, true positive rate; FP, false positive rate*.

**Table 5:** True positive and false positive rates for biased anchor with two additional biased item.

						**Free baseline**				**Fixed baseline**			
**Cell**	**L2N**	**L1N**	**ICC**	**Items**	**Size (%)**	**NLD**	**TP**	**NLD**	**FP**	**NLD**	**TP**	**NLD**	**FP**
127	50	2	0.10	2	1	19	0.03	13	0.06	325	0.29	324	0.30
128	50	2	0.10	2	5	14	0.03	9	0.16	181	0.01	156	0.04
139	50	2	0.20	2	1	15	0.04	18	0.06	236	0.03	203	0.03
140	50	2	0.20	2	5	16	0.05	9	0.14	216	0.03	152	0.05
151	50	2	0.30	2	1	21	0.04	11	0.04	231	0.02	204	0.01
152	50	2	0.30	2	5	137	0.28	134	0.31	197	0.03	127	0.07
129	50	5	0.10	2	1	14	0.03	16	0.04	193	0.03	189	0.03
130	50	5	0.10	2	5	9	0.02	1	0.55	121	0.07	27	0.33
141	50	5	0.20	2	1	19	0.03	18	0.05	164	0.02	159	0.04
142	50	5	0.20	2	5	27	0.02	2	0.44	102	0.09	18	0.35
153	50	5	0.30	2	1	30	0.03	13	0.04	159	0.04	123	0.03
154	50	5	0.30	2	5	23	0.01	1	0.42	112	0.06	29	0.37
131	50	25	0.10	2	1	17	0.04	3	0.30	64	0.09	18	0.26
132	50	25	0.10	2	5	16	0.02	0	1.00	0	0.79	0	1.00
143	50	25	0.20	2	1	19	0.04	3	0.32	58	0.09	14	0.31
144	50	25	0.20	2	5	5	0.04	0	1.00	0	0.79	0	1.00
155	50	25	0.30	2	1	15	0.04	2	0.44	43	0.13	8	0.44
156	50	25	0.30	2	5	7	0.02	0	1.00	0	0.85	0	1.00
133	100	2	0.10	2	1	25	0.05	23	0.04	230	0.02	226	0.02
134	100	2	0.10	2	5	16	0.06	5	0.29	230	0.02	146	0.07
145	100	2	0.20	2	1	29	0.04	24	0.03	235	0.03	218	0.02
146	100	2	0.20	2	5	22	0.09	12	0.29	191	0.03	91	0.11
157	100	2	0.30	2	1	39	0.03	23	0.04	243	0.04	222	0.02
158	100	2	0.30	2	5	31	0.11	7	0.25	197	0.04	87	0.12
135	100	5	0.10	2	1	28	0.02	16	0.05	168	0.03	150	0.04
136	100	5	0.10	2	5	21	0.03	0	0.82	70	0.15	8	0.60
147	100	5	0.20	2	1	26	0.02	14	0.05	175	0.02	112	0.06
148	100	5	0.20	2	5	21	0.04	0	0.73	63	0.16	3	0.63
159	100	5	0.30	2	1	20	0.04	18	0.07	157	0.04	106	0.05
160	100	5	0.30	2	5	11	0.04	0	0.79	52	0.15	0	0.70
137	100	25	0.10	2	1	16	0.04	0	0.55	43	0.15	4	0.53
138	100	25	0.10	2	5	12	0.03	0	1.00	0	0.98	0	1.00
149	100	25	0.20	2	1	19	0.05	1	0.63	27	0.19	2	0.63
150	100	25	0.20	2	5	15	0.03	15	0.03	0	0.98	0	1.00
161	100	25	0.30	2	1	16	0.04	0	0.76	28	0.18	2	0.76
162	100	25	0.30	2	5	18	0.02	0	1.00	0	1.00	0	1.00

*L2N, level-2 sample size; L1N, level −1 sample size; ICC, intraclass correlation; Items, number of biased items; NLD, count of negative log-likelihood difference test results; TP, true positive rate; FP, false positive rate*.

## Discussion

Breakthroughs in measurement invariance methods have made techniques available for testing measurement invariance across high numbers of groups with relatively small within group sample sizes. This is an important development, because until now the idea of testing whether different groups interpret survey questions similarly has been limited to a small number of groups with large sample sizes. Yet, the failure to adequately establish a common interpretation across groups is known to cause problems for interpretations of differences in latent means and relationships between latent variables. The method studied in this article to test measurement invariance, continuous indicator multilevel confirmatory factor analysis, is ideal for studying measurement invariance (i.e., cluster bias) across many groups. So far, it has been implemented using a constrained baseline approach, where the starting model has all factor loadings equal across levels and all level-2 residual variances fixed at 0. However, the growing literature on free baseline approaches suggests that a free baseline approach might have greater decision accuracy for bias detection. This article examined whether this is also the case for multilevel confirmatory factor analysis tests of measurement invariance. Indeed, support for a free baseline approach in a multilevel CFA setting was observed.

Overall, the power for the free baseline approach when the referent indicator was unbiased was 0.44. This was similar, albeit slightly higher, than the power for the constrained baseline approach under these conditions at 0.42. The real difference between the two methods when the referent indicator was unbiased was observed in the false positive rates. The overall false positive rate for the unbiased referent indicator under the free baseline was 0.04, which is acceptable. The false positive rate for the constrained baseline approach was unacceptably high at 0.14. When the referent indicator is unbiased, the free baseline approach should be preferred. Our first hypothesis, that a free (as opposed to a constrained or “fixed”) baseline approach would have an accuracy advantage in terms of true positive (power) and false positive (Type I error) was partially supported. While the free vs. fixed distinction was unrelated to the true positive rate, the free vs. fixed distinction was related to the false positive rate. Hypothesis 2 proposed that the improved decision accuracy under the free baseline approach would be greater under conditions that should lead to greater power and lower Type I error, such as increased ICC, level-2 sample size, level-1 sample size, number of biased items and bias magnitude. Indeed, several moderation effects were observed.

Exploration of the interaction between free vs. fixed baseline approach and L1N revealed that the constrained approach led to increased false positive rates at increased L1N. This is a theoretically interpretable result. The increased L1N is expected to magnify the power to detect the misspecified larger model under the constrained baseline approach, a misspecification that contravenes the assumption of the log-likelihood difference test and that is not present under the free baseline approach. The interaction between the free vs. fixed approach and the number of biased items also indicated that as the number of biased items increased from 1 to 2 items, the false positive rate increased. Once again, this can be considered theoretically consistent, because under the constrained baseline approach the inclusion of an additional misspecified item increases the degree of misspecification in the unrestricted model, and a correctly specified unrestricted model is required for the log-likelihood difference test. The final interaction between the free vs. fixed approach and the size of the bias indicated that as the bias increased so did the false positive rate when moving to the constrained baseline condition. Since increasing the size of the bias makes the misspecification more readily detectable, the violation of the assumption of the test procedure is once again more salient. Perhaps trumping all of these considerations, however, is the very high rate of negative log-likelihood difference test results under the constrained baseline set up. For all these reasons, the free baseline approach has more support when the anchor item is unbiased.

The measurement invariance literature has witnessed considerable research into the impact of violating the assumption of an unbiased indicator. This is because in practice, the assumption of an unbiased reference indicator might be easily violated. Therefore, we also examined what happens when the unbiased referent assumption is violated, since this assumption in practice can be difficult to check. Overall, when the referent indicator is biased, we saw that the false positive rate was unacceptably high regardless of whether a free or constrained baseline approach was used. The false positive rate for the free baseline approach was 0.25, and it was 0.29 for the fixed baseline approach. Moreover, the power was mediocre regardless of whether a free baseline approach or a fixed baseline approach was used. The power was 0.16 for the free baseline and 0.28 for the fixed baseline. These values make comparison of the advantages of one method over the other meaningless when the anchor item is biased. Instead, attention needs to be devoted to ensuring the referent item is unbiased. One approach that may be worthwhile considering is to first begin with the fully constrained model, and then examine modification indices to determine the item that is most likely to be unbiased. Once the unbiased item is identified, analyses can proceed according to the free baseline approach.

Researchers analyzing real data will need to make a series of decisions prior to the analysis and decisions during the analysis that will impact their ability to detect cluster bias. In terms of design considerations, this study reveals when the anchor item is unbiased, if the number of level-2 clusters is sufficiently large, increasing the level-1 sample size increases decision accuracy more than increasing level-2 sample size. In addition, the ICC sizes studied had a negligible impact on decision accuracy. This is fortunate, sample sizes may be more under the researcher's control than ICCs. This conclusion, of course, is conditional on an item being identified as unbiased for identification.

The limitations of this study relate primarily to the inability to be exhaustive in the simulation conditions, for instance, with a wider range of L2N. Our results also only focus on continuous variable measurement models, and conclusions may not apply for ordered categorical items where the free baseline model used here may not converge (early experimentation has indicated that the level-2 residual variances need to be fixed at zero for models to converge). Moreover, following this study there are still important questions to investigate. There are numerous other constrained baseline approaches that might be considered, and this study does not speak to these methods. For example, alternative constrained baseline methods may well perform better than the constrained baseline approach used here. These could include iterative freeing of residual variances based on modification indices, simultaneous freeing of all residual variances followed by determining significance with standard errors, or freeing residual variances one by one, and leaving the residual variance free for the item with the largest chi-square difference. Another avenue for research could be to examine whether the power of the cluster bias test increases when the likelihood ratio test distribution is adjusted to account for the level-2 residual variance test examining the boundary of the admissible parameter space (Stoel et al., 2006).

In summary, this study supports the free baseline approach when model assumptions are met. These might include situations where well developed psychometric instruments have been independently used in many different countries, and we know for instance, that similar items have corresponding high factor loadings in the different countries from independent research. In these instances, the lower false positive rate for the free baseline approach should lead to its adoption over the constrained baseline approach.

## Author contributions

The author confirms being the sole contributor of this work and approved it for publication.

### Conflict of interest statement

The author declares that the research was conducted in the absence of any commercial or financial relationships that could be construed as a potential conflict of interest.

## References

[B1] Asparouhov T. Muthén B. (2014). Multiple-group factor analysis alignment. Struct. Equ. Model. Multidiscip. J. 21, 495–508. 10.1080/10705511.2014.919210

[B2] Barendse M. Oort F. Werner C. Ligtvoet R. Schermelleh-Engel K. (2012). Measurement bias detection through factor analysis. Struct. Equ. Model. Multidiscip. J. 19, 561–579. 10.1080/10705511.2012.713261

[B3] Barendse M. T. Oort F. J. Garst G. J. (2010). Using restricted factor analysis with latent moderated structures to detect uniform and nonuniform measurement bias; a simulation study. AStA Adv. Stat. Anal. 94, 117–127. 10.1007/s10182-010-0126-1

[B4] Byrne B. M. Shavelson R. J. Muthen B. (1989). Testing for the equivalence of factor covariance and mean structures: the issue of partial measurement invariance. Psychol. Bull. 105, 456–466. 10.1037/0033-2909.105.3.456

[B5] Cham H. West S. G. Ma Y. Aiken L. S. (2012). Estimating latent variable interactions with nonnormal observed data: a comparison of four approaches. Multivariate Behav. Res. 47, 840–876. 10.1080/00273171.2012.732901 23457417 PMC3583564

[B6] Chan D. (1998). The conceptualization and analysis of change over time: an integrative approach incorporating longitudinal mean and covariance structures analysis (LMACS) and multiple indicator latent growth modeling (MLGM). Organ. Res. Methods 1, 421–483. 10.1177/109442819814004

[B7] Chen F. F. (2008). What happens if we compare chopsticks with forks? The impact of making inappropriate comparisons in cross-cultural research. J. Pers. Soc. Psychol. 95, 1005–1018. 10.1037/a0013193 18954190

[B8] Cheng C. Cheung M. W. L. Montasem A. (2014). Explaining differences in subjective well-being across 33 nations using multilevel models: universal personality, cultural relativity, and national income. J. Pers. 84, 46–58. 10.1111/jopy.12136 25234240

[B9] Cheung G. W. Rensvold R. B. (1999). Testing factorial invariance across groups: a reconceptualization and proposed new method. J. Manage 25, 1–27. 10.1177/014920639902500101

[B10] Cheung M. W. L. Leung K. Au K. (2006). Evaluating multilevel models in cross-cultural research: an illustration with social axioms. J. Cross Cult. Psychol. 37, 522–541. 10.1177/0022022106290476

[B11] Chun S. Stark S. Kim E. S. Chernyshenko O. S. (2016). MIMIC Methods for Detecting DIF Among Multiple Groups: exploring a New Sequential-Free Baseline Procedure. Appl. Psychol. Meas. 40, 486–499. 10.1177/0146621616659738 PMC5978634 29881065

[B12] Cieciuch J. Davidov E. Algesheimer R. Schmidt P. (2017). Testing for approximate measurement invariance of human values in the European Social Survey. Sociol. Methods Res. [Epub ahead of print]. 10.1177/0049124117701478

[B13] Cieciuch J. Shalom S. Davidov E. Schmidt P. Algesheimer R. (2014). Comparing results of an exact vs. an approximate (Bayesian) measurement invariance test: a cross-country illustration with a scale to measure 19 human values. Front. Psychol. 5:982. 10.3389/fpsyg.2014.00982 25249996 PMC4157555

[B14] Cohen J. (1988). Statistical Power Analysis for the Behavioral Sciences, 2nd Edn. Hillsdale, NJ: Routledge Academic.

[B15] Drasgow F. (1982). Biased test items and differential validity. Psychol. Bull. 92, 526–531. 10.1037/0033-2909.92.2.526

[B16] Drasgow F. (1984). Scrutinizing psychological tests: measurement equivalence and equivalent relations with external variables are the central issues. Psychol. Bull. 95, 134–135. 10.1037/0033-2909.95.1.134

[B17] Guenole N. (2016). The importance of isomorphism for conclusions about homology: a Bayesian multilevel structural equation modeling approach with ordinal indicators. Front. Psychol. 7:289. 10.3389/fpsyg.2016.00289 26973580 PMC4773641

[B18] Guenole N. Brown A. (2014). The consequences of ignoring measurement invariance for path coefficients in structural equation models. Front. Psychol. 5:980. 10.3389/fpsyg.2014.00980 25278911 PMC4166111

[B19] Jak S. Oort F. J. (2015). On the power of the test for cluster bias. Br. J. Math. Stat. Psychol. 68, 434–455. 10.1111/bmsp.12053 25776804

[B20] Jak S. Oort F. J. Dolan C. V. (2013). A test for cluster bias: detecting violations of measurement invariance across clusters in multilevel data. Struct. Equ. Modeling 20, 265–282. 10.1080/10705511.2013.769392

[B21] Jak S. Oort F. J. Dolan C. V. (2014). Measurement bias in multilevel data. Struct. Equ. Modeling 21, 31–39. 10.1080/10705511.2014.856694

[B22] Jang S. Kim E. S. Cao C. Allen T. D. Cooper C. L. Lapierre L. M. (2017). Measurement invariance of the satisfaction with life scale across 26 countries. J. Cross Cult. Psychol. 48, 560-576. 10.1177/0022022117697844

[B23] Joreskog K. G. Goldberger A. S. (1975). Estimation of a model with multiple indicators and multiple causes of a single latent variable. J. Am. Stat. Assoc. 70, 631–639.

[B24] Kim E. S. Kwok O. M. Yoon M. (2012a). Testing factorial invariance in multilevel data: a Monte Carlo study. Struct. Equ. Model. Multidiscip. J. 19, 250–267. 10.1080/10705511.2012.659623

[B25] Kim E. S. Yoon M. Lee T. (2012b). Testing measurement invariance using MIMIC: likelihood ratio test with a critical value adjustment. Educ. Psychol. Meas. 72, 469–492. 10.1177/0013164411427395

[B26] Maas C. J. M. Hox J. J. (2005). Sufficient sample sizes for multilevel modeling. Methodology 1, 86–92. 10.1027/1614-1881.1.3.86

[B27] Mellenbergh G. J. (1989). Item bias and item response theory. Int. J. Educ. Res. 13, 127–143. 10.1016/0883-0355(89)90002-5

[B28] Meredith W. (1993). Measurement invariance, factor analysis and factorial invariance. Psychometrika 58, 525–543. 10.1007/BF02294825

[B29] Millsap R. E. (2012). Statistical Approaches to Measurement Invariance. London: Routledge.

[B30] Muthén B. Khoo S. T. Gustafsson J. E. (1997). Multilevel Latent Variable Modeling in Multiple Populations. Unpublished Technical Report. Available Online at: http://www.statmodel.com

[B31] Muthén B. O. (1991). Multilevel factor analysis of class and student achievement components. J. Edu. Meas. 28, 338–354. 10.1111/j.1745-3984.1991.tb00363.x

[B32] Muthén B. O. (1994). Multilevel covariance structure analysis. Sociol. Methods Res. 22, 376–398. 10.1177/0049124194022003006

[B33] Muthén L. K. Muthén B. O. (1998-2017). Mplus User's Guide, 8th Edn. Los Angeles, CA: Muthén & Muthén.

[B34] Navas-Ara M. J. Gómez-Benito J. (2002). Effects of ability scale purification on identification of DIF. Eur. J. Psychol. Asses. 18, 9–15. 10.1027//1015-5759.18.1.9

[B35] Oort F. J. (1998). Simulation study of item bias detection with restricted factor analysis. Struct. Equ. Modeling Multidiscip. J. 5, 107–124. 10.1080/10705519809540095

[B36] Paxton P. Curran P. J. Bollen K. A. Kirby J. Chen F. (2001). Monte Carlo experiments: design and implementation. Struct. Equ. Modeling 8, 287–312. 10.1207/S15328007SEM0802_7

[B37] Rabe-Hesketh S. Skrondal A. Pickles A. (2004). Generalized multilevel structural equation modelling. Psychometrika 69, 167–190. 10.1007/BF02295939

[B38] Ryu E. (2014). Factorial invariance in multilevel confirmatory factor analysis. Br. J. Math. Stat. Psychol. 67, 172–194. 10.1111/bmsp.12014 23682861

[B39] Satorra A. Bentler P. M. (2001). A scaled difference chi-square test statistic for moment structure analysis. Psychometrika 66, 507–514. 10.1007/BF02296192 PMC2905175 20640194

[B40] Sorbom D. (1974). A general method for studying differences in factor means and factor structure between groups. Br. J. Math. Stat. Psychol. 27, 229–239. 10.1111/j.2044-8317.1974.tb00543.x

[B41] Stark S. Chernyshenko O. S. Drasgow F. (2006). Detecting differential item functioning with confirmatory factor analysis and item response theory: toward a unified strategy. J. Appl. Psychol. 91, 1292–1306. 10.1037/0021-9010.91.6.1292 17100485

[B42] Stoel R. D. Garre F. G. Dolan C. van den Wittenboer G. (2006). On the likelihood ratio test in structural equation modeling when parameters are subject to boundary constraints. Psychol. Methods 11, 439–455. 10.1037/1082-989X.11.4.439 17154756

[B43] Tay L. Woo S. E. Vermunt J. K. (2014). A conceptual framework of cross-level Isomorphism: psychometric validation of multilevel constructs. Organ. Res. Methods 17, 77–106. 10.1177/1094428113517008

[B44] Vandenberg R. J. Lance C. E. (2000). A review and synthesis of the measurement invariance literature: suggestions, practices, and recommendations for organizational research. Organ. Res. Methods 3, 4–70. 10.1177/109442810031002

[B45] van de Schoot A. G. J. Schmidt P. De Beuckelaer A. (2015). Measurement Invariance. Lausanne: Front. Media. 10.3389/fpsyg.2015.01064 PMC4516821 26283995

[B46] Wang W. Tay L. Drasgow F. (2013). Detecting differential item functioning of polytomous items for an ideal point response process. Appl. Psychol. Meas. 37, 316–335. 10.1177/0146621613476156

